# A structural basis for the functional differences between the cytosolic and plastid phosphoglucose isomerase isozymes

**DOI:** 10.1371/journal.pone.0272647

**Published:** 2022-09-01

**Authors:** Juan Jiao, Fei Gao, Jie Liu, Zongyang Lv, Cuimin Liu

**Affiliations:** 1 Department of Clinical Laboratory, 7th Medical Center of Chinese PLA General Hospital, Beijing, China; 2 State Key Laboratory of Plant Cell and Chromosome Engineering, Institute of Genetics and Developmental Biology, Beijing, China; 3 Department of Research and development, Beijing IPE Center for Clinical Laboratory CO., Ltd, Beijing, China; 4 Department of Biochemistry and Structural Biology, University of Texas Health Science Center at San Antonio, San Antonio, TX, United States of America; Weizmann Institute of Science, ISRAEL

## Abstract

Phosphoglucose isomerase (PGI) catalyzes the interconversion between glucose-6-phosphate (G6P) and fructose-6-phosphate (F6P), thereby regulating sucrose synthesis in plant cells. In general, plants contain a pair of PGI isozymes located in two distinct compartments of the cell (cytosol and plastid) with differences in both the primary structure and the higher-order structure. Previously, we showed that the activity of cytosolic PGI (PGIc) is more robust (activity, thermal stability, substrate turnover rate, etc.) than that of the plastid counterpart (PGIp) in multiple organisms, including wheat, rice, and *Arabidopsis*. The crystal structures of apoTaPGIc (an isotype cytosol PGIc in *Triticum aestivum*), TaPGIc-G6P complex, and apoTaPGIp (an isotype plastid PGIp in *Triticum aestivum*) were first solved in higher plants, especially in crops. In this study, we detailed the structural characteristics related to the biochemical properties and functions of TaPGIs in different plant organelles. We found that the C-terminal domains (CTDs) of TaPGIc and TaPGIp are very different, which affects the stability of the dimerized enzyme, and that Lys213_TaPGIc_/Lys193_TaPGIp_ and its surrounding residues at the binding pocket gateway may participate in the entrance and exit of substrates. Our findings provide a good example illuminating the evolution of proteins from primary to higher structures as a result of physical barriers and adaptation to the biochemical environment.

## Introduction

During glycolysis, phosphoglucose isomerase (PGI, EC 5.3.1.9) catalyzes the reversible isomerization of glucose-6-phosphate (G6P) and fructose-6-phosphate (F6P). Two kinds of separately evolving PGI isoenzymes, plastidic (PGIp) and cytosolic (PGIc) PGIs, have been previously detected in different sublocations in higher plants [1]. The biochemical properties of these PGIs, such as catalyzing activity, thermal stability, and substrate turnover rate in vitro, are dramatically different [1]. However, in vivo studies reveal that PGIc and PGIp are both involved in diverse mechanisms of glucose synthesis (sucrose, starch) [2, 3]. Many works have reported that PGIs are vital to plants. In the chloroplast, PGIp catalyzes the initial step of starch synthesis from the primary photosynthetic product F6P. The G6P intermediate generated in this step is ultimately converted into starch. According to previous studies, *pgip* mutants exhibit starch synthesis deficiency and a late flowering phenotype in *Arabidopsis* [4, 5]. Meanwhile, in the cytosol, PGIc is involved in sucrose synthesis and glycolysis. Specifically, it regulates the balance between the triose phosphate and hexose phosphate pools in plant cells. PGIc gene knockdown mutants in *Arabidopsis* lead to low sucrose content in leaves and impaired photosynthesis, and PGIc gene knockout is nonviable (lethal) for homozygous T-DNA insertion [6]. In summary, PGIs play important roles in carbon cycling, energy fluxing, and the development of plants.

In mammals, PGIs exhibit a type of isomerase as well as anomerase activity [7, 8], and act as neuroleukins [9, 10], differentiation and maturation mediators [11], and autocrine motility factors (AMFs) [12, 13], all of which are involved in extracellular activities. Comparatively, plant PGIs exhibit only isomerase activity, with no evidence of either anomerase or extracellular activities. Considering the multiple functions of PGIs depending on their 3-D structures, many PGI architectures have been resolved and investigated in recent decades. Based on these structures, the mechanisms of inhibitor binding (E4P, 5PA), substrate binding (G6P, F6P), and substrate conversion [14–18] have been well described.

In our previous work [1], three PGI crystal structures (TaPGIc, TaPGIc-G6P and TaPGIp) were solved for the first time in higher plants. We established that the TaPGI isozymes have different properties, including catalytic activity and substrate binding affinity, which can probably be ascribed to their location in different organelles. It was also shown that the incorporation of TaPGIc in the chloroplast of an *Arabidopsis thaliana pgip* mutant leads to starch overaccumulation, as well as increased CO_2_ assimilation, plant biomass, and even seed yield productivity. However, it remains unclear what factors make PGI isozymes so different and why TaPGI has higher activity, stability, and productivity than TaPGIp. Herein, this study is extended to illuminate the structural details of TaPGIc and TaPGIp and to explore the key points that make this pair of isomerase twins different. In this work, we found that a key Lys residue (Lys213TaPGIc/Lys193TaPGIp) and some structural elements (the C-terminal domain (CTD) and the helix-loop-helix motif (HLHM)) are the factors making these isozymes so distinct.

## Results

### Structural basis for the distinct dimeric structures of TaPGIp and TaPGIc

**Fig 1:**
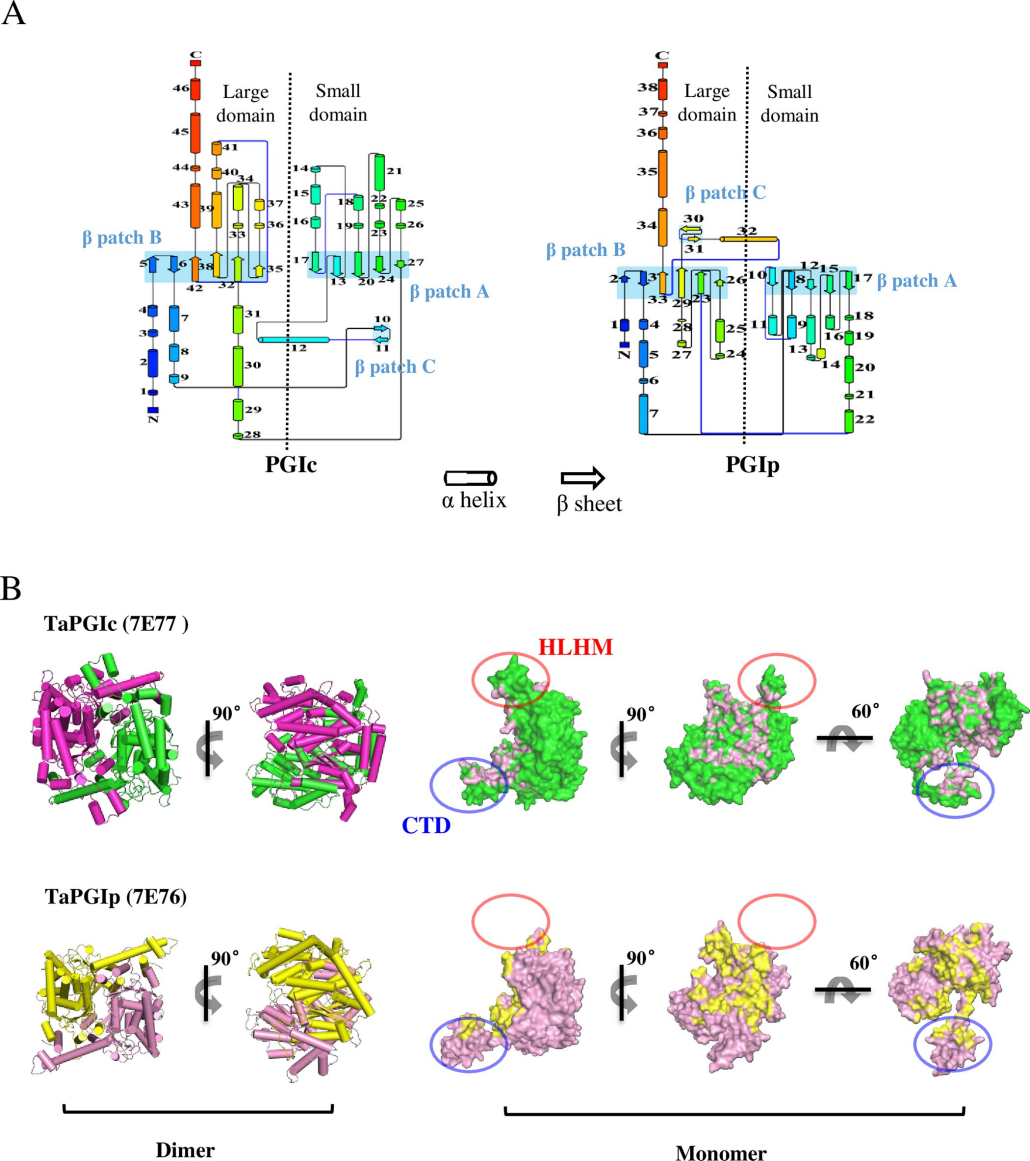
The crystal structures of TaPGIc and TaPGIp. **A.** Second structure topology map of TaPGIc and TaPGIp in their crystal structures. TaPGIc has 46 basic secondary structure elements, and TaPGIp has 38 similar elements. Both TaPGIs contain three groups of β-strands, termed β patches (A, B, and C), and each β patch is composed of various numbers of β-strands. The cylinder indicates the α helix, and the broad arrow indicates the β-strand. **B.** Overall structures of the TaPGIc and TaPGIp complexes (dimer conformation and monomer conformation). The architecture of TaPGIc is shown in magenta and green, and TaPGIp is shown in yellow and pink. The dotted circles show two different regions between TaPGIc and TaPGIp, named “HLHM” (highlighted by red circle) and “CTD” (highlighted by blue circle). PGIc HLHM is from 444aa to 466aa. PGIc CTD is from 510aa to 567aa, and PGIp CTD is from 467aa to 557aa.

In our previous work, the dimeric structures of TaPGIp and TaPGIc were first reported in plants. We described some general different structural features between this pair of isozymes [1], but the detailed structural divergence between these two proteins has not been described. Here, we present a detailed structural analysis of these two isomerase twins. In the 3D dimeric architectures of TaPGIp and TaPGIc (Fig 1A and 1B), each protomer consists of two core domains. The “small domain” comprises five parallel β strands (labeled β patch A) and their surrounding helices, whereas the “large domain” contains six parallel/antiparallel β sheets (labeled β patch B) and their interacting helices. Each β sheet patch has a strong hydrophobic core and is flanked by helices. The strong hydrophobic interactions at the interface between the β sheets and α helices of the small and large domains stabilize the protomer, resulting in a stable conformation of the whole PGI enzyme. These two hydrophobic interfaces comprise a large number of hydrophobic residues that are conserved in TaPGIc and TaPGIp (S1 Fig).

The “ring/ball” shaped wheat TaPGIc dimeric architecture is similar to that of some well-described cytosolic PGIs in other species, such as *Homo sapiens* PGIc (HoPGIc), *Escherichia coli* PGIc (EcPGIc), *Mus musculus* PGIc (MsPGIc), and *Toxoplasma gondii* PGIc (ToPGIc), and it is consistent with the high identity (>40%) in amino sequences [14–19] (S2 Fig). In contrast, wheat TaPGIp, the unique structure in plastids, exhibits a distinct “rhombus/diamond” shape architecture and shares only very low identity to TaPGIc (22%), HoPGIc (24%), and EcPGIc (23%) (S2 Fig). At first glance, the major differences between TaPGIc and TaPGIp structures are attributed to two structural elements, the HLHM and the CTD (Fig 1B). The HLHM (444aa to 466aa) is present in TaPGIc and other reported PGIs (such as HoPGIc, EcPGIc, MsPGIc, and ToPGIc) but is almost missing in TaPGIp (S2 Fig). The C-terminal extension domain is a common feature of TaPGIp, TaPGIc, and other PGIs [11–14, 16]; however, the CTD of TaPGIp (467aa-557aa) is much longer than that of TaPGIc (510aa-567aa) and other PGIs (approximately 60 aa length) (S2 Fig). These distinct structural elements in TaPGIp may contribute to its stretched architecture (Fig 1B).

### The CTD is vital for maintaining the dimeric structure and activity

As mentioned above, TaPGIc and TaPGIp have two distinct structural elements: the HLHM and the CTD. Previously, we first reported that the dimeric state and activity of PGIs are seriously affected by a series of TaPGIc mutants with different CTD truncations. The TaPGIc_ΔCTDα45α46_ mutant results in monomers with no activity, while the TaPGIc_ΔCTDα46_ mutant leads to protein aggregation with abolished activity, probably due to the exposure of the hydrophobic surface [1].

**Fig 2:**
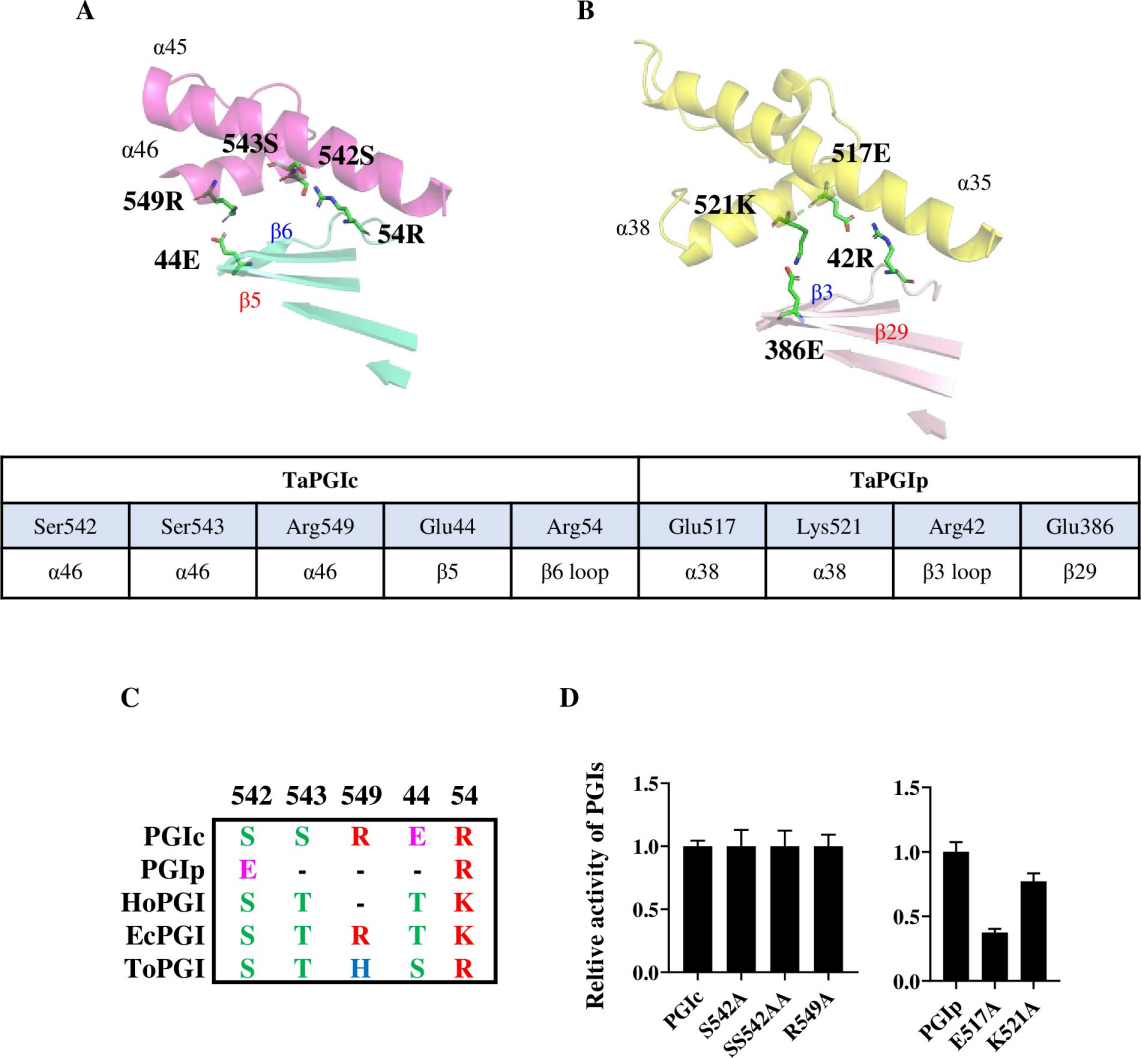
The C-terminal helix affected the quaternary structure and activity of TaPGIs. **A.** Residues affecting the quaternary structure and activity of TaPGIc at the CTD. Three residues (Ser542, Ser543, and Arg549) from the last C-terminal helix and 2 residues (Glu44 and Arg54) from β patch B and its extension loop in TaPGIc affect the quaternary structure and activity of TaPGIc. **B.** Residues affecting the quaternary structure and activity of TaPGIp at the CTD. Two residues (Glu517 and Lys521) from the last CTD and 2 residues (Arg42 and Glu386) from β patch B and its extension loop in TaPGIp affect the quaternary structure and activity of TaPGIp. **C.** Alignment of residues involved in the interaction described in B from different organisms. The residues Glu44, Arg54, Ser542, Ser543, and Arg549 of TaPGIc are not conserved among HoPGIc (from *Homo sapiens*), EcPGIc (from *Escherichia coli*), ToPGIc (from *Toxoplasma gondii*), and TaPGIp. **D.** The relative activities of TaPGIs and their mutation variants. The sites mutated in TaPGIs are indicated as subscripts. n≥3. Error bars indicate the standard deviation.

In the apoTaPGIc model (Figs 2A and S3), CTD_TaPGIc_ (α45 and α46, 510aa–567aa) interacts with β patch B (β5, β6-loop), the HLHM (444aa-466aa), and the α39 helix (426aa–443aa) via chemical bonds, including H bonds, salt bridges, and hydrophobic interactions. Because the HLHM is missing in the apoTaPGIp model, CTD_TaPGIp_ (α36-α38, 467aa-557aa) interacts only with β patch B (β3-loop, β29) and the α32 helix (405aa-423aa). It has already been reported that two hydrogen bonds with Ser542-Arg54 and Ser543-Arg54, as well as a salt bridge of Arg549-Glu44, participate in the interaction of the CTD and β patch B in TaPGIc [1] (Fig 2A). Here, we found that only salt bridges between Glu517-Arg42 and Lys521-Glu386 participate in the contacts between CTD and β patch B in TaPGIp (Fig 2B).

The TaPGI CTDs interact similarly with β patch B: in TaPGIc, Ser542, Ser543, and Arg549 are located in the α46 helices, and Arg54 and Glu44 are located in the β5 and β6 extended loops, respectively; in TaPGIp, Glu517 and Lys521 are located in the α38 helices, and Arg42 and Glu386 are located in the β patch B β3 extended loop and β29, respectively (Fig 2A and 2B). Alignment with reported PGIs (TaPGIs, HoPGIc, EcPGIc, ToPGIc) indicates that these residues are not conserved but maintain similar polarity (Fig 2C). Activity assays showed that S542A, SS542AA, and R549A variants exhibited similar activity to native TaPGIc, whereas in TaPGIp, only a single Ala substitution in E517A and K521A variants may lead to greatly reduced activity compared to that of native TaPGIp (Fig 2D, S1 Table). Together with our previous results that only opposite charge replacements in the S542D, S543D, and SS542DD variants resulted in disordered quaternary structure and a severe decrease in TaPGIc’s activity (S1 Table) [1], this finding implied that the difference in CTD and the deletion of HLHM seriously affected the interactions between CTD_TaPGIp_ and its partner protomer, resulting in poorer stability and activity of TaPGIp than that of TaPGIc.

### The active sites of TaPGIc and TaPGIp

**Fig 3:**
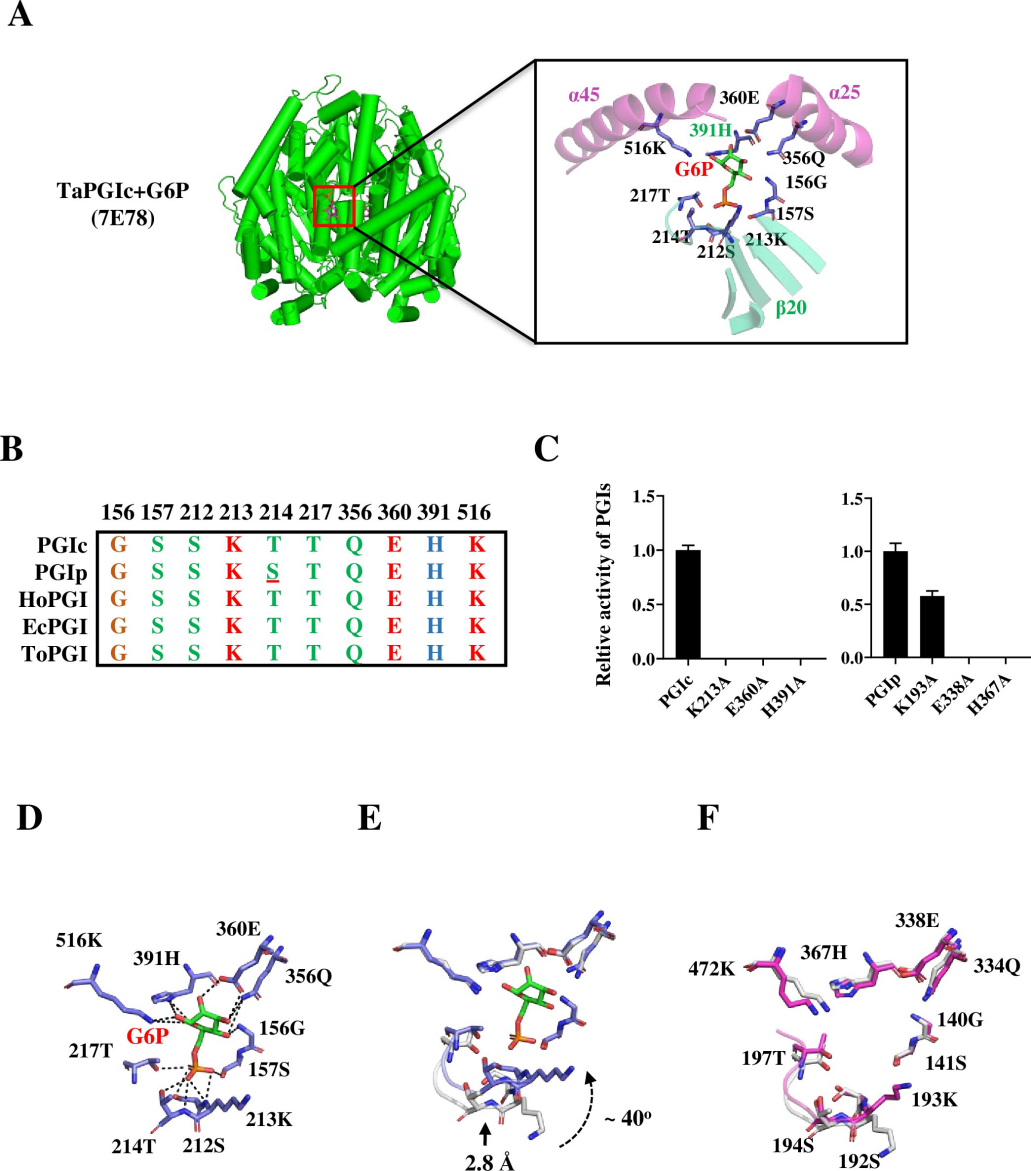
The active sites of TaPGIc and TaPGIp. **A.** The activity sites of the TaPGIs are located at the interface of the two subunits of the complex binding with G6P. Two residues from two separate α-helixes, 6 residues from β patch A and its extension loop, and a residue from the partner subunit compose the active sites of the TaPGIs. **B.** The alignment of PGI active sites from different organisms. The residues Gly156, Ser157, Ser212, Lys213, Thr214, Thr217, Gln356, Glu360, His391, and Lys516 in TaPGIc are conserved among TaPGIp, HoPGIc (from *Homo sapiens*), EcPGIc (from *Escherichia coli*), and ToPGIc (from *Toxoplasma gondii*). **C.** The relative activities of TaPGIs and their mutation variants. The sites mutated in TaPGIs are indicated as subscripts. n≥3. Error bars indicate the standard deviation. **D-F.** Enlarged views showing G6P and its binding sites. D shows the closed state (binding substrate state) TaPGIc active sites, G6P in green, and active sites in blue. The Glu360 OE2 atom contacts the O2 and O3 atoms (sugar ring) at 2.8 Å and 2.9 Å. The His391 ND1 atom contacts the O1 and O5 atoms (sugar ring) at 3.6 Å and 3.1 Å. The Lys516 NZ atom contacts the O1 and O5 atoms (sugar ring) at 2.8 Å and 4.8 Å. The atoms of Ser157 OG, Thr217 OG1, Ser212 OG, Thr 214 OG1, Thr 214 N, Lys213 N contact the phosphate group of G6P within ~ 3 Å; E shows the open state (unloaded substrate state) of TaPGIc active sites (in gray) superposed with the closed state of active sites (in blue); F shows the open state of TaPGIp active sites (in magenta) superposed with the open state of TaPGIc active sites (in gray).

The complex structure of TaPGIc with G6P was also previously determined at a resolution of 2.05 Å [1]. The electron density corresponding to bound G6P and active site residues is clear in both protomers of the dimer (Figs 3A and S4A). The active site of TaPGIc is located in the cleft between the large and small domains of each protomer and is also close to the interface between the two protomers, as described in previous crystallographic studies [14]. Analysis of the G6P binding pocket reveals that many residues are involved in substrate binding and catalysis. The active site of TaPGIc consists of Gly156, Ser157, Ser212, Lys213, Thr214, Thr217, Gln356, Glu360 and Lys516 residues from one protomer and an extra His391 residue from the other protomer, which highlights the importance of the dimeric architecture for maintaining the catalytic activity of TaPGIc (Fig 3A). Notably, the TaPGIc catalytic pocket is highly hydrophilic (S4B Fig), and therefore can favorably bind to hydrophilic substrates such as glucose-6-phosphate, which has four hydroxyl groups and one phosphate group. In the TaPGIc-G6P complex model, the constellation of threonine and serine residues (Ser157, Ser212, Lys213, Thr214, and Thr217) located at one end of the active site are responsible for interacting with the phosphate group of G6P. Specifically, the Ser157, Ser212, Thr214, and Thr217 side chains and the Lys213 and Thr214 backbone amide groups form H bonds with the G6P phosphate group. At the other end of the active site, a polar patch formed by Gln356, Glu360, His391, and Lys516 interacts with the oxygen atoms from the hydroxyl groups of G6P through H bonds (Fig 3A and 3D). The active site residues are strictly conserved from lower organisms (*Escherichia coli* and *Toxoplasma gondii*) to higher organisms (*Homo sapiens* and *Triticum aestivum*), except for a Thr/Ser substitution in TaPGIp (Fig 3B). Mutation variant activity assays showed that Glu360_TaPGIc_/Glu338_TaPGIp_ and His391_TaPGIc_/His367_TaPGIp_ can seriously affect the activity of both TaPGIc and TaPGIp. However, Lys213_TaPGIc_ and Lys193_TaPGIp_ may have diverse roles in catalysis; the mutation K213A in TaPGIc resulted in almost no activity, while the K193A mutation in TaPGIp reduced the activity of the PGI by only half (Fig 3C, S1 Table).

By superposing the TaPGIc-G6P model on the apoTaPGIc and apoTaPGIp models, the mechanism of G6P binding and isomerization may be elucidated (Fig 3D–3F). Once the G6P substrate is loaded onto the open state (substrate free) active site, the main chains of Lys213_TaPGIc_ and Thr214_TaPGIc_ are influenced by interactions with the phosphate group of G6P. Consequently, the extension loop of β20 (β20-loop) is shifted 2.8 Å by this interaction, and the side chain of Lys213_TaPGIc_ rotates approximately 40 degrees (Fig 3E). Finally, the active site of TaPGIc adopts the closed state (substrate loaded). On the other hand, the residues His391, Lys516, and Glu360, which contact the sugar ring of G6P, exhibit almost no shifts in position compared to the substrate-free conformation. In the complex of TaPGIc with G6P, the Glu360 OE2 atom, His391 ND1 atom, and Lys518 NZ atom contact the O1, O2, O3 and O5 atoms of the sugar ring. It is worth noting that the ND1 atom of His391 is 3.1 Å from the O5 atom and 3.6 Å from the O1 atom in the sugar ring; the NZ atom of Lys518 is 2.9 Å and 4.8 Å from the O1 atom and O5 atom; and the Glu360 OE2 atom contacts the O2 and O3 atoms at distances of 2.8 Å and 2.9 Å, respectively (Fig 3D). This has implications for the mechanism of sugar ring opening (discussed below). In general, after G6P loading, the substrate binding pocket gateway closes and provides a relatively secluded environment for isomerization.

Another novel finding is that compared to apoTaPGIc (open state), the positions of most active site residues of apoTaPGIp (open state) do not shift, except for a considerable rotation of the side chain of Lys193_TaPGIp_, whose conformation is close to that in the TaPGIc-G6P model (closed state) (Fig 3E and 3F). This implies that the TaPGIp binding pocket gateway may have some defects in substrate binding or catalysis.

### Lys213 participates in substrate entrance and exit at the binding pocket gateway

**Fig 4:**
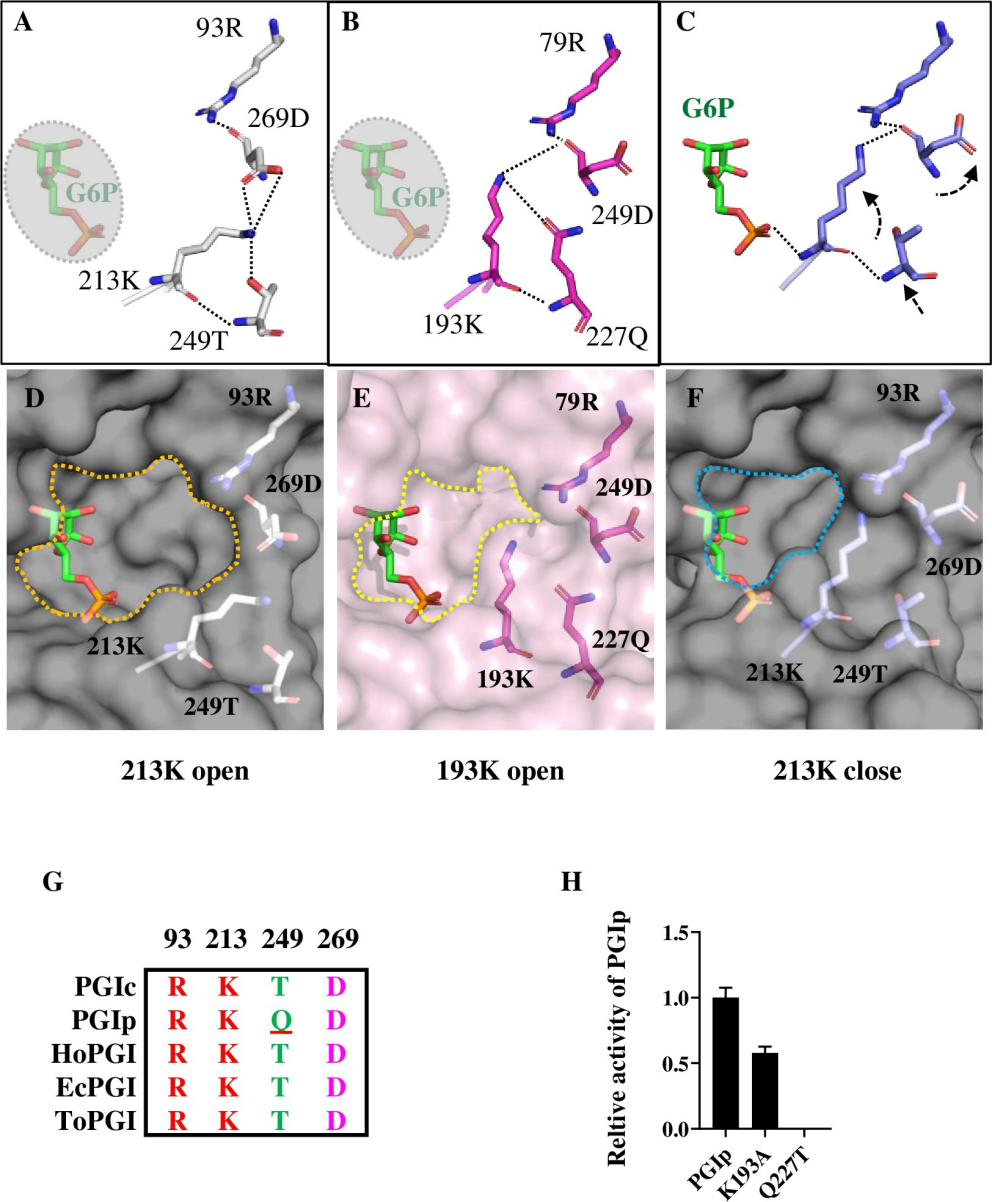
The residue Lys213_TaPGIc_/Lys193_TaPGIp_ participates in substrate entrance and exit. **A-C.** Lys213_TaPGIc_/Lys193_TaPGIp_ and its interacting residues surrounding the substrate binding pocket of TaPGIs. A shows Lys213_TaPGIc_ and its interacting residues (in white) in the open state of the binding pocket of TaPGIc. B shows Lys213_TaPGIc_ and its interacting residues (in blue) in the closed state of the binding pocket of TaPGIc. C shows Lys193_TaPGIp_ and its interacting residues (in magenta) in the open state of the binding pocket of TaPGIp, and G6P is in green. The black dotted arrows indicate the shift of some particular residues at the binding pocket gateway. **D-F.** Lys213_TaPGIc_/Lys193_TaPGIp_ and its interacting residues superposed on the surface model of the substrate binding pocket of TaPGIs. Dotted lines show the substrate binding pocket gateway. **G.** The alignment of Lys213_TaPGIc_/Lys193_TaPGIp_ and its interacting residues from different organisms. The residues of Arg93, Lys213, Thr249, and Asp269 in TaPGIc are conserved among HoPGIc (from *Homo sapiens*), EcPGIc (from *Escherichia coli*), ToPGIc (from *Toxoplasma gondii*), and TaPGIp. **H.** The relative activities of TaPGIs and their mutation variants. The sites mutated in TaPGIs are indicated as subscripts. n≥3. Error bars indicate the standard deviation.

Based on the results presented above, it is likely that the movement of Lys213_TaPGIc_ is vital for substrate binding or catalysis. In fact, Lys213 is involved in completely different sets of interactions in the open- and closed-state conformations of the substrate binding pocket. In the TaPGIc model (open state), the main chain of Lys213 interacts with the main chain of Thr249, and the side chain of Lys213 is engaged in H bonding interactions with the side chains of Thr249 and Asp269 (Figs 4A and S4C and S4D). Similarly, in the TaPGIp model (open state), the main chains of Lys193 interact with the main chains of Gln227, and the side chain of Lys193 engages in H bonding with the side chain of Gln227 and the main chain of Asp249 (Figs 4B and S4D). After G6P is loaded in the active site (closed state, in the TaPGIc-G6P model), the H bond contact between the main chain of Lys213 and the phosphate group of G6P induces an obvious movement of Lys213, resulting in an obvious shift in the Thr249 position, as well as a 90-degree rotation of the Asp269 side chain (Figs 4C and S4C and S4D). In addition, the side chain of Lys213 engages in H bonding only with the main chain of Asp269, which may stabilize the conformation of Lys213 and the binding pocket gateway in the closed-state conformation of the substrate binding pocket. Notably, the residues Lys213_TaPGIc_/Lys193_TaPGIp_, Thr249_TaPGIc_/Gln227_TaPGIp_, and Asp269_TaPGIc_/Asp249_TaPGIp_ have also been identified in other reported PGI primary structures, and they are fully conserved in different organisms, except for a Thr/Gln replacement in TaPGIp (Fig 4G). The superposition of 213K_TaPGIc_/193K_TaPGIp_ and its interacting residues with the corresponding surface models shows us that they are located at the gateway of the substrate-binding pocket (Fig 4D–4F). Another surprising finding is that the binding pocket gateway of apoTaPGIp (open state) is similar to that of TaPGIc-G6P (closed state) (Fig 4E). Activity assays showed that the mutation Q227T (mimicking Thr249 in TaPGIc) did not improve but instead abolished the activity of TaPGIp (Fig 4H, S1 Table). The above results suggested that Lys213_TaPGIc_/Lys193_TaPGIp_ and the β20-loop play vital roles in substrate binding; however, the mechanism of substrate entrance and exit at TaPGI binding pockets is still unclear.

## Discussion

PGI is well known as a type of isomerase that participates in glycolysis reactions in bacteria, animals, and plants. In plants, PGI has evolved into two branches, one of which is located in the plastid (chloroplast) and the other in the cytosol. Previously, we showed that plastidic and cytosolic PGIs are clustered into separate phylogenetic branches in both lower and higher plants, which indicates that the evolution of PGIs is an early event in plant evolutionary history [1]. By BLASTing TaPGIc, TaPGIp and other cytosolic PGIs deposited in the Protein Data Bank (PDB, https://www.pdbus.org/), it was shown that TaPGIp shares less than 24% identity with TaPGIc and other PGIcs in amino sequences, whereas the identity between TaPGIc and other PGIcs is no less than 40% (S2 Fig). Moreover, TaPGIp and TaPGIc exhibit dramatic differences in enzyme properties; for example, TaPGIc has much higher activity, thermal stability, and substrate turnover rate [1], and TaPGIc is more sensitive to erythrose 4-phosphate (E4P, a PGI inhibitor) than TaPGIp, with IC50_E4P_ values of TaPGIc and TaPGIp of 0.13 (± 0.01) and 0.76 (± 0.05) mM, respectively (S5 Fig).

Herein, this work explored the key factors in the primary and tertiary structure of TaPGIp and TaPGIc related to the dramatic variation in their functions and biochemical properties. Three novel plant PGI crystal structures (apoTaPGIp, apoTaPGIc, and TaPGIc-G6P complex) were determined, which are the first 3D architectures of PGI in plants. In addition, the apoTaPGIp structure is the first 3D architecture of plastid PGI. Our results may provide an opportunity to systematically investigate the function and organelle differentiation of PGIs in relation to structure. Similar to other reported PGI structures (HoPGIc, MsPGIc, ToPGIc, and EcPGIc), TaPGIs have a dimeric structure that consists of two protomers, and each protomer contains two hydrophobic β-sheet cores surrounded by alpha helixes (Figs 1A and S1). This conformation provides a relatively solid scaffold for the whole enzyme, but maintains some flexibility between the two hydrophobic core domains. TaPGIc, like other cytosolic PGIs, exhibits a “ring/ball” architecture, whereas TaPGIp has a “rhombus/diamond” structure.

In general, the most obvious differences in TaPGIp and TaPGIc structures are associated with the CTD and HLHM elements (Fig 1B), which were never mentioned in other previous reports until the TaPGIp structure was resolved. According to primary structure alignment, the CTDs and HLHMs are not conserved in different species, especially TaPGIp (S2 Fig). CTD_TaPGIp_ is much longer than other cytosolic PGI CTDs (including TaPGIc), with only 8% identity versus 20% identity between the cytosolic PGI CTDs (including TaPGIc) (S2 Fig). CTD_TaPGIc_ has three interfaces (CTD-β patch B, CTD-HLHM, and CTD-α39 Helix) interacting with its partner protomer in the TaPGIc model (Figs 2A and S3A). Truncated CTD_TaPGIc_ seriously affects the dimeric state and activity of TaPGIc [1]. Furthermore, as the HLHM is absent in TaPGIp, CTD_TaPGIp_ has only two interfaces (CTD-β patch B and CTD-α32 helix) that interact with its partner protomer (Figs 2B and S3B), which may greatly weaken the interaction between the two protomers of the TaPGIp dimeric architecture. This was proven by the substitution activity assay of the residues that form H bonds or salt bridges located in the CTD (Fig 2A–2D, S1 Table). Therefore, the above results provide new insight into CTD structural and biochemical functions by comparing PGIs from two organelles (TaPGIc and TaPGIp).

Although, at the current resolution, the electron density could not distinguish a distinct conformer for the sugar ring of G6P and suggests a mixed population of boat and chair forms, it was that the residues Gln356, Glu360, Lys516, His391, Ser157, Ser212, Lys213, Thr214, and Thr217 compose the active site of TaPGIc (Fig 3A); it is not surprising that residues at the active site are conserved from *E*. *coli* to higher organisms and vital for enzyme activity (Fig 3B and 3C, S1 Table). The structure of the substrate-binding pockets of TaPGIs has an exquisite confirmation in which each residue at the active site has a distinct role in substrate binding and catalysis. The substrate catalysis mechanism was well described previously; Glu357_MsPGIc_ is used as the base catalyst in the isomerization reaction, and His388_MsPGIc_ is used as an acid catalyst in ring opening in mouse [16] and other species [14, 17, 18]. By adopting a similar mechanism, TaPGIc in complex with the G6P structure implicated Glu360_TaPGIc_ as the base catalyst in the isomerization reaction and His391_TaPGIc_ as an acid catalyst in ring opening. Although Lys516 and His391 are equidistant from O5, a relatively long distance (4.8 Å and 6.6 Å respectively in two active site) between the amino group of Lys516 and the ring oxygen atom shows that it is unlikely to abstract a proton from or donate a proton to O5 and that His391 is much better placed (3.1 Å or 4.2 Å respectively in two active site) for this role. Lys516 forms a close contact with the C1 hydroxyl group and O1 atom, which suggests that it might participate in the ring-opening step, which involves protonation of C1 (to give F6P) or C2 (to give G6P). However, together with other reported works [16–18], due to the close contact between Glu360 and carbon atoms C1 (3.7 Å) and C2 (3.3 Å), it is more likely that Glu360 participates in the double bond generation between C1 and C2 and mediates the isomerization reaction in the TaPGIc. Other active site residues of Ser157, Ser212, Lys213, Thr214, and Thr217 are responsible for anchoring the phosphate group of G6P (Fig 3D). In addition, the residue of Arg274_TaPGIc_ responding to the residues Arg272 in rabbits and mice, which plays an important role in substrate catalysis [16, 18], is 4.2 Å from G6P, suggesting that it may also help to stabilize the intermediates during sugar ring opening in higher plants. It is worth mentioning that Lys516 is involved in both binding to the CTD and catalysis at the active site. It seems to be an active site sensor that senses the subtle changes in dimeric structure mediated by CTD.

Our structures showed some other interesting findings. For instance, there is an obvious shift (2.8 Å) of the β20-loop (containing the main chains of Ser212, Lys213, and Thr214) and a 40° rotation of a side chain of Lys213 during the active site turn to the closed state conformation (G6P loaded) from the open state conformation (G6P free) (Fig 3D and 3E), which seems unlikely to be caused by crystal packing because a similar conformation can be found in some inhibitor- or substrate-bound structures, such as MsPGI-E4P [16]. These movements lead to closure of the active site cavity mouth at the surface of the TaPGIc structure [1]. Therefore, we supposed that Lys213 may participate in substrate entrance and exit the binding pocket. The conformation of TaPGIp substrate-binding pockets may give us a chance to investigate this process in more depth. The active site of TaPGIs has a similar conformation in their apo state (open state), and there is little movement on the backbone and side chains of the active site, except for the Lys193_TaPGIp_ residue, whose side chain is significantly shifted compared to the Lys213_TaPGIc_ side chain (Fig 3F). Intriguingly, Lys213/Lys193 and a group of conserved residues of Arg93/Arg79, Asp269/Asp249, and Thr249/Gln227 form a H-bond interacting patch located at the gateway of the substrate-binding pocket. Once G6P is loaded into the active site of TaPGIc, the phosphate group of G6P contacts the main chain of Lys213 and pulls Asp269 and Thr249 forward to the substrate, resulting in reestablishment of the interaction network of these residues, followed by shrinking of the active site cavity mouth (Fig 4A–4G). It is worth noting that in the TaPGIc and TaPGIp active site models, Lys193_TaPGIp_ has a conformation distinct from that of Lys213_TaPGIc_ and has a notable replacement of Thr249_TaPGIc_ with Gln227_TaPGIp_. As the side chain of glutamine is much larger than that of threonine, there is a steric hindrance effect, causing Lys193_TaPGIp_ to adopt a “medium” conformation (a medium state between the open and closed states of Lys213_TaPGIc_). This may partly explain why TaPGIp has some defects in substrate turnover rate and catalytic efficiency. Substitution activity assays showed that the activity of K213A_TaPGIc_ was almost abolished, but K193A_TaPGIp_ activity reached as much as half that of native TaPGIp (Fig 3C, S1 Table). This implied that replacement with alanine at Lys213 induces failure of gateway closing and substrate catalysis in TaPGIc, but the large side chain of Gln227 may partly rescue the gateway closing failure in the substitution variant of K193A in TaPGIp. However, when we replaced glutamine with threonine at TaPGIp (mimicking threonine at 249 in TaPGIc), no activity could be detected (Fig 4H, S1 Table). This result suggested that a simple substitution cannot improve TaPGIp activity by mimicking TaPGIc and that not only Lys213/193 and its contact residues but also other factors may be involved in substrate exit and entrance at the substrate binding pocket gateway.

In higher plants, PGIp-mediated glycolysis is a light-independent reaction implicated in plant photosynthesis (Calvin cycle). Considering the low efficiency of Rubisco, other enzymes involved in the light-independent reactions of plants may face extremely low evolutionary selection pressure, making the CTD and HLHM of PGIs mutation hotspots during the evolution process. Therefore, the properties of PGIps change significantly, with lower thermal stability, lower activity, lower turnover rate, etc. By comparing TaPGIc and such different TaPGIp, we have a chance to glance at some novel details in the substrate binding and isomerization process of PGIs that have never been considered in previous reports. Our work provided some clues for studying the substrate binding and isomerization mechanism of PGIs in plants, and more effort should be made in biochemistry and genetics to confirm the structural findings. In summary, this work highlights the structural and phylogenetic mechanisms of PGI isoenzyme pairs. Based on current and previous results [1], plant photosynthesis may be enhanced by replacing or increasing the robustness of isoenzymes in the Calvin cycle.

## Supporting information

S1 Fig The β-sheet patches in large and small domain in TaPGIs. **A.** The β-sheet patch A in TaPGIc, contains β-sheet 13, 17, 20, 24, 27. The hydrophobic residues V149, V150, V152, L183, F185, L186, L207, V208, V209, V210, V211, M244, I245, A246, V247, A264, F265, A266 are compose of the hydrophobic β patch A. **B.** The β-sheet patch A in TaPGIp, contains β-sheet 8, 10, 12, 15, 17. The hydrophobic residues I133, L134, I161, F163, I164, V188, I189, V190, I191, G222, V223, A224, I225, F245, P246 are compose of the hydrophobic β patch A. **C.** The trans-reverse β-sheet patch B in TaPGIc, contains β-sheet 5, 6, 32, 35, 38, 42. The hydrophobic residues A43, V48, F49, L50, A337, A339, I340, L341, P342, I380, F381, F407, I408, G409, V410, L472, F474, L475, L476 are compose of the hydrophobic β patch B. **D.** The trans-reverse β-sheet patch B in TaPGIp, contains β-sheet 2, 3, 23, 26, 29, 33. The hydrophobic residues L36, F37, V38, M316, V317, V318, L319, V357, F380, F381, V382, F384, I385, V387, V430, V432 are compose of the hydrophobic β patch B.
(PDF)

S2 Fig Amino Sequences alignment of TaPGIc and TaPGIp with deposited PGIs in PDB. The PGIs PDB ID used in the alignment analysis (by ClustalW method) are as follow: TaPGIp, 7E76; TaPGIc, 7E77; EcPGIc, 3NBU; ToPGIc, 3UJH; MsPGIc, 2CXR; HoPGIc, 1JLH.
(PDF)

S3 Fig The interaction between HLHM and CTD holding the TaPGIs tertiary structure. **A.** The contacts between the HLHM-α39_TaPGIc_ (426aa-466aa) element and the CTD_TaPGIc_ help the TaPGIc to form dimer complex. The interacted residues listed below. Hy, hydrophobic interactions; H, H bond; S, Salt bridge. **B.** The contacts between the HLHM-α32_TaPGIp_ (405aa-423aa) helix and the CTD_TaPGIp_ help the TaPGIp to form dimer complex. The interacted residues listed below. Hy, hydrophobic interactions; H, H bond; S, Salt bridge.
(PDF)

S4 Fig The conformation of G6P and Active site residues with their electric density. **A.** The conformation of G6P and active site residues the in TaPGIc-G6P complex with very good electric density. **B.** Surface electrostatic potential model of the catalytic pocket of TaPGIc-G6P complex. **C.** The open/closed state conformation of the extension loop of β20 (Ser212, Lys213, Thr214, Thr217) in apoTaPGIc/TaPGIc-G6P complex and with very good electric density. **D.** The conformation of Lys213TaPGIc/Lys193TaPGIp in open/closed state with electric density.
(PDF)

S5 Fig Inhibition of TaPGIc (A) and TaPGIp (B) activity by Erythrose-4-phosphate (E4P). TaPGIs activity was assayed with various concentrations E4P (0.05 to 2.5 mM) and 2 mM F6P. Each point is the mean of at least three independent measurements. And the IC_50_ of TaPGIs to E4P was determined using dose-response inhibition method with Excel software.
(PDF)

S1 Table The activity data sheet of TaPGIs mutated variants. All variants of PGI were list in this chart for the activity assay. It also showed which variant were cited by this work or our previous work, and each activity assay experiment was repeated at least three times.
(PDF)

S1 Appendix (DOCX)
