# Supplementary figures and images for: A structural basis for the functional differences between the cytosolic and plastid phosphoglucose isomerase isozymes
Source: PLoS One. 2022 Sep 1;17(9):e0272647. doi: 10.1371/journal.pone.0272647 (PMC9436075; doi:10.1371/journal.pone.0272647)

TaPGIc

TaPGIp

A

B

 $\beta$  patch A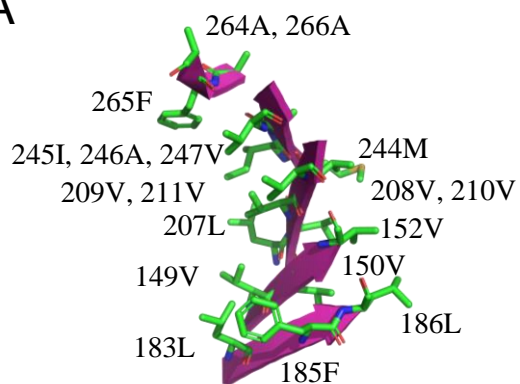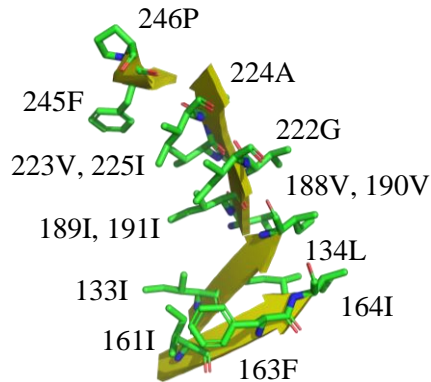

C

D

 $\beta$  patch B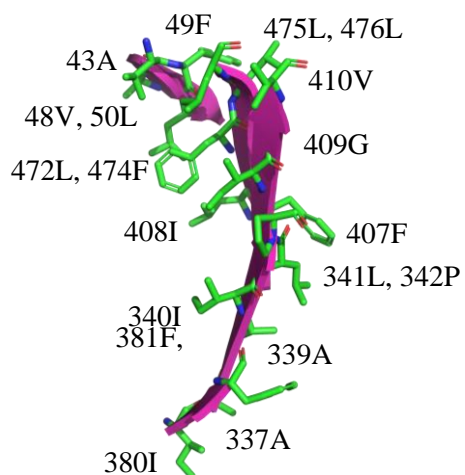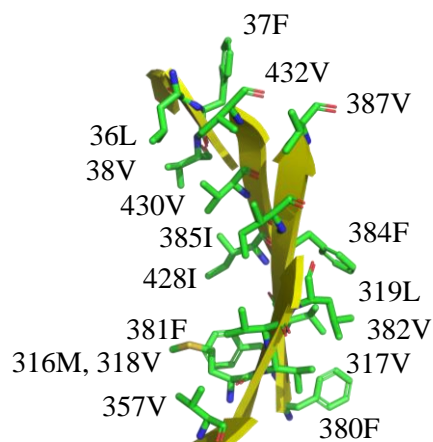

Supplement: S1 Fig — A. The β-sheet patch A in TaPGIc, contains β-sheet 13, 17, 20, 24, 27. The hydrophobic residues V149, V150, V152, L183, F185, L186, L207, V208, V209, V210, V211, M244, I245, A246, V247, A264, F265, A266 are compose of the hydrophobic β patch A. B. The β-sheet patch A in TaPGIp, contains β-sheet 8, 10, 12, 15, 17. The hydrophobic residues I133, L134, I161, F163, I164, V188, I189, V190, I191, G222, V223, A224, I225, F245, P246 are compose of the hydrophobic β patch A. C. The trans-reverse β-sheet patch B in TaPGIc, contains β-sheet 5, 6, 32, 35, 38, 42. The hydrophobic residues A43, V48, F49, L50, A337, A339, I340, L341, P342, I380, F381, F407, I408, G409, V410, L472, F474, L475, L476 are compose of the hydrophobic β patch B. D. The trans-reverse β-sheet patch B in TaPGIp, contains β-sheet 2, 3, 23, 26, 29, 33. The hydrophobic residues L36, F37, V38, M316, V317, V318, L319, V357, F380, F381, V382, F384, I385, V387, V430, V432 are compose of the hydrophobic β patch B. (PDF) [file pone.0272647.s001.pdf]

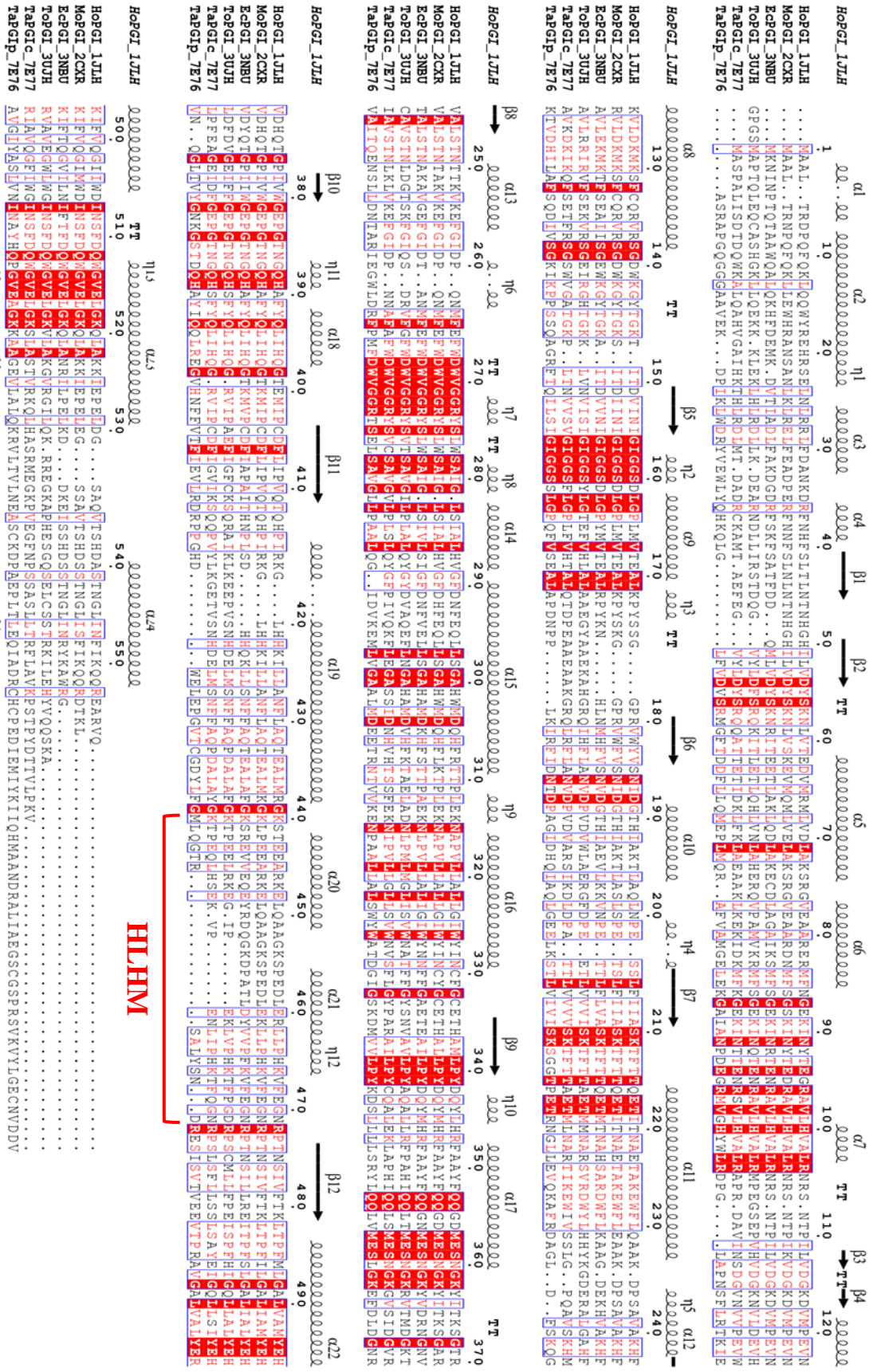

Supplement: S2 Fig — The PGIs PDB ID used in the alignment analysis (by ClustalW method) are as follow: TaPGIp, 7E76; TaPGIc, 7E77; EcPGIc, 3NBU; ToPGIc, 3UJH; MsPGIc, 2CXR; HoPGIc, 1JLH. (PDF) [file pone.0272647.s002.pdf]

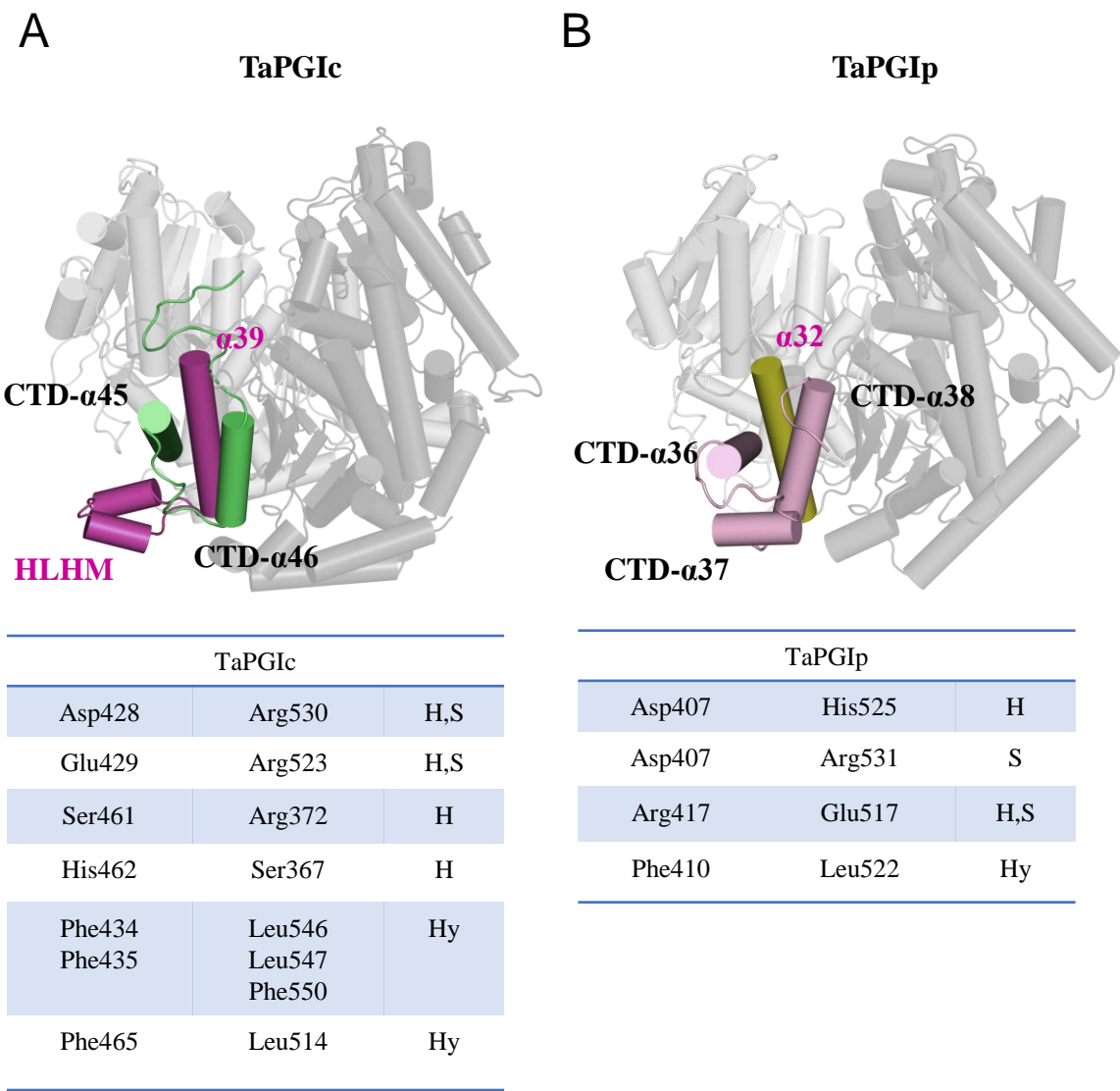

Supplement: S3 Fig — A. The contacts between the HLHM-α39TaPGIc (426aa-466aa) element and the CTDTaPGIc help the TaPGIc to form dimer complex. The interacted residues listed below. Hy, hydrophobic interactions; H, H bond; S, Salt bridge. B. The contacts between the HLHM-α32TaPGIp (405aa-423aa) helix and the CTDTaPGIp help the TaPGIp to form dimer complex. The interacted residues listed below. Hy, hydrophobic interactions; H, H bond; S, Salt bridge. (PDF) [file pone.0272647.s003.pdf]

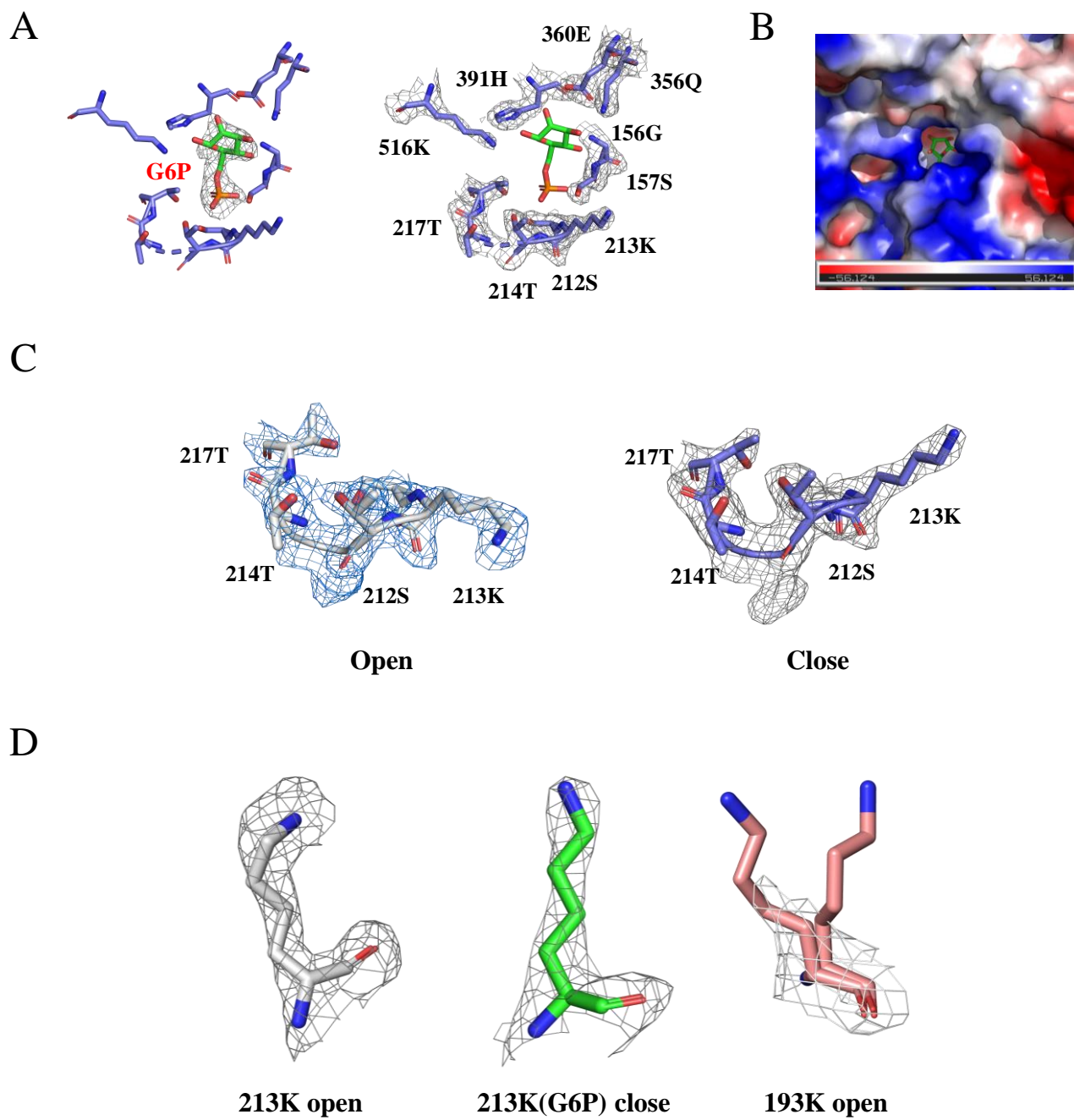

Supplement: S4 Fig — A. The conformation of G6P and active site residues the in TaPGIc-G6P complex with very good electric density. B. Surface electrostatic potential model of the catalytic pocket of TaPGIc-G6P complex. C. The open/closed state conformation of the extension loop of β20 (Ser212, Lys213, Thr214, Thr217) in apoTaPGIc/TaPGIc-G6P complex and with very good electric density. D. The conformation of Lys213TaPGIc/Lys193TaPGIp in open/closed state with electric density. (PDF) [file pone.0272647.s004.pdf]

A

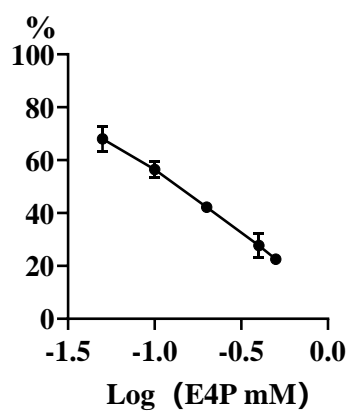 $\text{TaPGIc}_{\text{IC}_{50}} = 0.13 \pm 0.01 \text{ mM}$ 

B

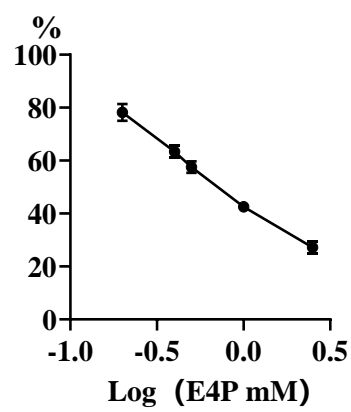 $\text{TaPGIp}_{\text{IC}_{50}} = 0.76 \pm 0.05 \text{ mM}$

Supplement: S5 Fig — TaPGIs activity was assayed with various concentrations E4P (0.05 to 2.5 mM) and 2 mM F6P. Each point is the mean of at least three independent measurements. And the IC50 of TaPGIs to E4P was determined using dose-response inhibition method with Excel software. (PDF) [file pone.0272647.s005.pdf]
