# Supplementary material for: A structural basis for the functional differences between the cytosolic and plastid phosphoglucose isomerase isozymes
Source: PLoS One. 2022 Sep 1;17(9):e0272647. doi: 10.1371/journal.pone.0272647 (PMC9436075; doi:10.1371/journal.pone.0272647)
Supplement: S1 Table — All variants of PGI were list in this chart for the activity assay. It also showed which variant were cited by this work or our previous work, and each activity assay experiment was repeated at least three times. (PDF) [file pone.0272647.s006.pdf]

S1 Table.

Variants	isoenzyme	location	Activity（Units/mg）	Note
TaPGIc (WT)	TaPGIc	-	756±35	In this work & [1]
TaPGIp (WT)	TaPGIp	-	88.2±5	In this work & [1]
S542A	TaPGIc	CTD	753±33	In this work & [1]
SS542AA	TaPGIc	CTD	763±42	In this work
S542D	TaPGIc	CTD	480±18	[1]
SS542DD	TaPGIc	CTD	322±18	[1]
R549A	TaPGIc	CTD	743±43	In this work & [1]
E517A	TaPGIp	CTD	33.0±2.2	In this work
K521A	TaPGIp	CTD	67.9±4.1	In this work
K213A	TaPGIc	Active Site	No activity	In this work
Q356A	TaPGIc	Active Site	60.8±2.7	In this work & [1]
E360A	TaPGIc	Active Site	No activity	In this work
H391A	TaPGIc	Active Site	No activity	In this work & [1]
K193A	TaPGIp	Active Site	50.8±2.9	In this work
Q227T	TaPGIp	Active Site	No activity	In this work
Q334A	TaPGIp	Active Site	7.3±0.41	In this work
E338A	TaPGIp	Active Site	No activity	In this work
H367A	TaPGIp	Active Site	No activity	In this work

Note: the variants activity data were cited by this work or our previous work [1].
