# Supplementary material for: A structural basis for the functional differences between the cytosolic and plastid phosphoglucose isomerase isozymes
Source: PLoS One. 2022 Sep 1;17(9):e0272647. doi: 10.1371/journal.pone.0272647 (PMC9436075; doi:10.1371/journal.pone.0272647)
Supplement: S1 Appendix — (DOCX) [file pone.0272647.s007.docx]

**Experimental Procedures**

**Protein expression and purification**

The protein samples used in this study were prepared as described in Gao’s article. [1]. Briefly, the CDS (cDNA sequences) of mature PGI proteins in wheat cDNA was first amplified by polymerase chain reaction (PCR) using specific primers. The PCR products were ligated to the pHUE expression vector to express PGI with a His-Ub tag. To obtain various mutants of PGIs, a standard mutagenesis protocol was used [1], and the plasmids were verified by DNA sequencing before incorporation into *E. coli* BL21 (DE3) cells.

BL21 cells containing different expression plasmids were grown to an OD600 of 1–1.2 and induced with 0.1 mM isopropyl-β-D-galactopyranoside (IPTG) at 16°C overnight. Subsequently, the cells were harvested and resuspended in lysis buffer (50 mM NaH_2_PO_4_, 300 mM NaCl, pH 8.0) and then disrupted by ultrasonication (Sonicator S-4000, Misonix). After centrifugation at 20,000 × g and 4°C, the soluble fractions were collected and eluted through a Ni-NTA column (GE Healthcare) using lysis buffer supplemented with 300 mM imidazole as the mobile phase. To remove the His-Ub tag, the purified proteins were pooled and digested overnight with ubiquitin proteinase (U) at 4°C. Finally, the PGI proteins were purified by size exclusion chromatography (Superdex 200, GE Healthcare) using a storage buffer comprised of 20 mM Tris-HCl (pH 7.5) and 100 mM NaCl. All purified proteins were concentrated to approximately 10 mg/mL, snap-frozen with liquid nitrogen, and stored at −80°C until further use. TaPGIs and their variants are dimeric complexes, as detected by the AF4 system.

**Protein activity assay**

The activity of PGIs was measured using an NADPH-coupled assay according to our previous work with some modifications [1]. Solutions (100 μL) of 62.5 mM glycylglycine buffer (pH 7.4), 5 mM fructose-6-phosphate, 5 mM MgCl_2_, 1 mM NADP, and 0.5-unit glucose-6-phosphate dehydrogenase were premixed well, and 0.3 mM PGIs or its point mutations were added to the mixed buffer. The reaction was allowed to proceed for 3 min. The absorbance of these solutions at 340 nm was recorded every 10 s using a Synergy HTX spectrophotometer (BioTek, US).

To determine the IC50 values of TaPGIc and TaPGIp, E4P concentrations were varied between 0.05 and 2.5 mM, and the concentration of fructose-6-phosphate was set to 2 mM. The IC50 values were calculated based on dose–response inhibition tests using Excel software.

**Structural analysis**

The coordinates of the crystal structures were obtained from the RCSB Protein Data Bank (PDB) under accession codes 7E76 (apo TaPGIp), 7E77 (apo TaPGIc), and 7E78 (TaPGIc with G6P). Visual structural analysis was carried out using PyMOL, and other deposited PGIs were used for amino sequence alignment: 3NBU (EcPGIc, *Escherichia coli*), 3UJH (ToPGIc, *Toxoplasma gondii*), 2CXR (MsPGIc, *Mus musculus*), and 1JLH (HoPGIc, *Homo sapiens*).

**Protein information**

The UniProt (www.uniprot.org/) accession IDs of mature TaPGIs in this work are A0A3B5ZN99 (TaPGIc) and A0A3B6LNA8 (TaPGIp).
